# Chinese Emergency Event Recognition Using Conv-RDBiGRU Model

**DOI:** 10.1155/2020/7090918

**Published:** 2020-05-21

**Authors:** Haoran Yin, Jinxuan Cao, Luzhe Cao, Guodong Wang

**Affiliations:** College of Police Information Engineering & Cyber Security, People's Public Security University of China, Beijing, China

## Abstract

In view of the weak generalization of traditional event recognition methods, the limitation of dependence on field knowledge of expert, the longer train time of deep neural network, and the problem of gradient dispersion, the neural network joint model, Conv-RDBiGRU, integrated residual structure was proposed. Firstly, text corpus is preprocessed by word segmentation and stop words processing and uses word embedding to form the matrix of word vectors. Then, local semantic features are extracted through convolution operation, and deep context semantic features are extracted through RDBiGRU. Finally, the learned features are activated by softmax function and the recognition results are output. The novelty of work is that we integrate residual structure into recurrent neural network and combine these methods and field of application. The simulation results show that this method improves precision and recall of Chinese emergency event recognition, and the *F*-value is better than other methods.

## 1. Introduction

As a manifestation of information, an event is defined as the objective fact that specific people and objects interact with each other at a specific time and place [1]. The Internet is full of all kinds of disorderly emergency event news, which is intermingled with other news, and these other news will hinder clear cognition of users about emergency event and relevant researchers' work in classification and storage [2], so how to realize emergency event recognition in the network is one of the problems that need to be solved at present. Event recognition is an important basis for event extraction. Event recognition refers to extracting information that has uniform format, structured and event interesting to users from unstructured text information and makes corresponding classification of events [3].

At present, there are three main methods of event recognition, which are based on pattern matching, machine learning, and deep learning. In pattern matching, the feature template of the corresponding event is designed in advance. In [4], it proposes a novel Event-Adaptive Concept Integration algorithm to estimate the effectiveness of semantically related concepts by assigning different weights to them and harnesses the related concepts which are discriminatively matched for a target event to obtain good performance. In [5], enhancing the discovered patterns with linguistic information (morphological and POS categories) is considered to recognize criminal events against people from Spanish newspapers. In [6], a set of methods for creation of such vocabularies is proposed, such as a vocabulary of subordination models, a vocabulary of event triggers, and a vocabulary of Frame Elements, in Russian and other languages using Google Books NGram Corpus to match Russian event. In [7], an event template extraction approach which incorporates structured knowledge bases is presented, and the approach outperforms similar methods that do not use structured knowledge bases. However, the method based on pattern matching relies on expert domain knowledge, and the model generalization is not strong. In machine learning, we mainly focus on feature discovery and how to construct classifier to transform event recognition into classification problem. In [8], variational mode decomposition (VMD) and a newly developed weighted online sequential extreme learning machine (WOSELM) are integrated to detect and classify the power quality events (PQEs) in real-time by using different advanced classifiers. In [9], it proposes an improved TF-PDF algorithm to recognize emergency event according to the relatively stable combination between hot words. The discretely labeled data is trained with HMM and Conditional Random Field classifiers and reports a substantial improvement in performance of event recognition [10]. On the basis of extracting traditional features [11], the features of semantic role SR are added and then CRFs are used to recognize event. With the development of deep learning technology, in the field of emergency event recognition, artificial neural network has obtained more and more attention and has been successfully applied in this field to solve practical problems. In [12], the DCNNs-LSTM model is proposed to realize emergency event recognition of cohesive language Uygur. In [13], the classifier which combines support vector machine with radial basis function neural network to improve the reliability of an event recognition result is proposed. In [14], a recurrent convolutional neural network model with attention mechanism based on language model is proposed, which mainly solves the problem of polysemy and multievent sentence recognition. In [15], an anomaly event recognition model that uses intratrace and intertrace context vectors with long short term memory networks to overcome the challenge of online anomaly event recognition in cyber-physical systems is introduced. In [16], a dynamic masked attention network model is proposed to capture richer contextual information so that leading experimental results are obtained in the multievent extraction task. In [17], a novel approach is proposed to recognize volcano-seismic events based on fully connected DNNs. This approach can efficiently capture complex relationships of volcano-seismic data and achieve better classification performance with faster convergence when compared to classical models. The model employs a Bidirectional Long Short Term Memory (BLSTM) neural network and multilevel attention mechanism for event extraction and achieves good results in biomedical event recognition [18]. In [19], it presents a hybrid-supervised DBN, which combines the unsupervised and supervised learning to improve performance of event recognition and realize recognition of other relevant elements in the event.

**Figure 1:**
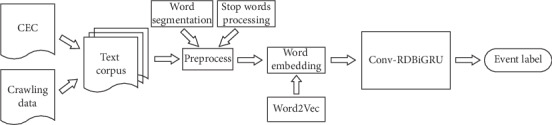
Overall learning scheme.

In the current field of event recognition, traditional methods rely on expert knowledge and artificial design features. These methods have a high accuracy in the professional field, but the generalization ability is not good in the event recognition of an open field. The use of the deep learning model for event recognition have higher portability, but there are many parameters that need to be adjusted in the process of model training, and as the number of iterations increases, the training gradient is prone to dispersion, and the single neural network framework has certain limitations. In order to tackle the abovementioned problems, a neural network model Conv-RDBiGRU (Convolution-Residual Deep Bidirectional Gated Unit) was proposed in this paper, and the overall learning scheme is shown in Figure 1. In the model, extracting local information feature of text through convolution operation, capturing deep context feature information of text corpus using DBiGRU part, and then using the residual structure to change the framework structure that data source for this network layer are only from previous layer in the traditional plain network. The changed framework structure introduces a design that is similar to “shortcut” type [20]. The source data input skips multiple hidden layers and is added directly to the data output section. This method can introduce more reference information to the output data, and, at the same time, make up the influence of vanishing gradient along with the network deepened, so as to improve Chinese emergency event recognition effect.

## 2. Related Work

### 2.1. Preprocess

Text statements are extracted from the corpus by using regular expression. It is a common idea of English event recognition to extract features of words in sentences through neural network, find out existing trigger words, and classify them [21]. However, for some languages, such as Chinese, there is no natural segmentation in Chinese, so, at first, word segmentation is required, and word segmentation tools are used to process text. Common word segmentation tools include jieba, Stanford, CRF++, and thulac [22]. The Word2Vec model proposed by MIKOLOV et al. is used to train the text corpus after word segmentation to obtain the word vector [23]. The training methods include Skip-Gram and CBOW (Continuous Bag of Words) [24].

### 2.2. Convolution

There are three common structures, convolutional layer, pooling layer, and full connection layer, in convolutional neural network (CNN). After the convolution layer, the maximum pooling operation will be taken to reduce the dimension of the output result for capturing significant local features, and then the next process outputs the result through the fully connected operation. However, in this paper, the text corpus is sequence text with context-dependent information. It will lose sequence information of word with each other and destroy the sequence features if next step adopts the pooling operation after convolution. So, in the model, after using the convolution operation, the next step will abandon the relevant operation about the pooling layer and fully connected layer. The local features that extracted convolution operation are directly inputted into the next layer network structure for extracting deeper context semantic features.

### 2.3. RNN Variant

Theoretically, RNN (Recurrent Neural Network) can cope with the context sequence information of random distance, but, in the actual application process, traditional RNN structure do not have sufficient ability to capture long distance information features. The robustness is not good so that it cannot achieve the expected training effect, and in the hidden layer, the error produced by the abovementioned information increases constantly as the count of transmission increases. This situation causes the problem of gradient dispersion and explosion in the process of training. In order to solve these problems, RNN's unit structure has produced many variants, including LSTM (Long Short Term Memory), GRU (Gated Recurrent Unit), and SRU (Simple Recurrent Unit).

**Figure 2:**
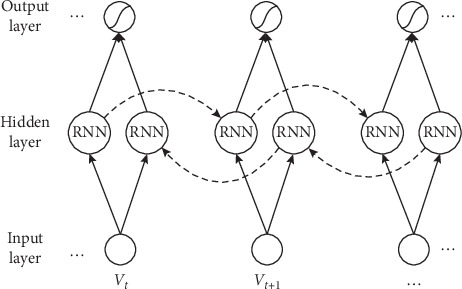
BiRNN structure model.

The usually mentioned RNN model is a unidirectional transmission mechanism to transfer the previous moment sequence information, namely, the UniRNN (Unidirectional Recurrent Neural Network), which can only predict the output of the next moment through the abovementioned information features. However, in the practice process, the information of some moments is not only related to the above sequence information but also related to the following sequence information. For example, there is such a scene, “My computer is down, so I want to__a new computer.” If we only observe statements before the underline, it cannot judge the next step what “I want to__.” However, clearly, the next step is that “I want to buy a new computer” if we can observe information after the underline, so the probability will be much bigger to fill in the word “buy” on the underline. Therefore, in order to solve the shortage of UniRNN, a BiRNN (Bidirectional Recurrent Neural Network) is proposed, as shown in Figure 2.

BiRNN is composed of a forward $\overset{⟶}{\text{RNN}}$ and a backward $\overset{⟶}{\text{RNN}}$. The input layer is the word vector matrix *V*={*v*_1_, *v*_2_,…, *v*_*t*_}. The hidden layer contains the unit structure of RNN. The structure can be LSTM, GRU, SRU, and other RNN variants. The output layer is determined by the two direction output state in RNN.

### 2.4. Residual Network

**Figure 3:**
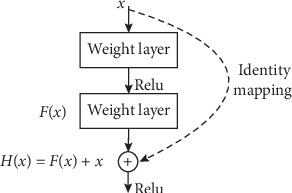
Residual unit.

Theoretically, for neural networks, the richer features can be learned from the multilayer abstract information if the more layers are stacked and the network is deeper. However, the gradient will vanish in the train process with the deepening of the network so that train time of whole network becomes longer, and it is difficult to converge and the network performance degrades. In order to solve these problems, a residual network structure is proposed and the structure unit is shown in Figure 3.

Residual unit is generally composed of two mappings, the identity mapping *x* and the residuals mapping *F*(*x*). For the general network without residual structure, it is expected to use *F*(*x*) as the feature to activate and output. Now, the residual structure is integrated, so it is expected to use *H*(*x*) to fit. The output result contains the input information *x* itself, which is transformed from the original input by identity mapping. It is a richer information feature.

## 3. Model

**Figure 4:**
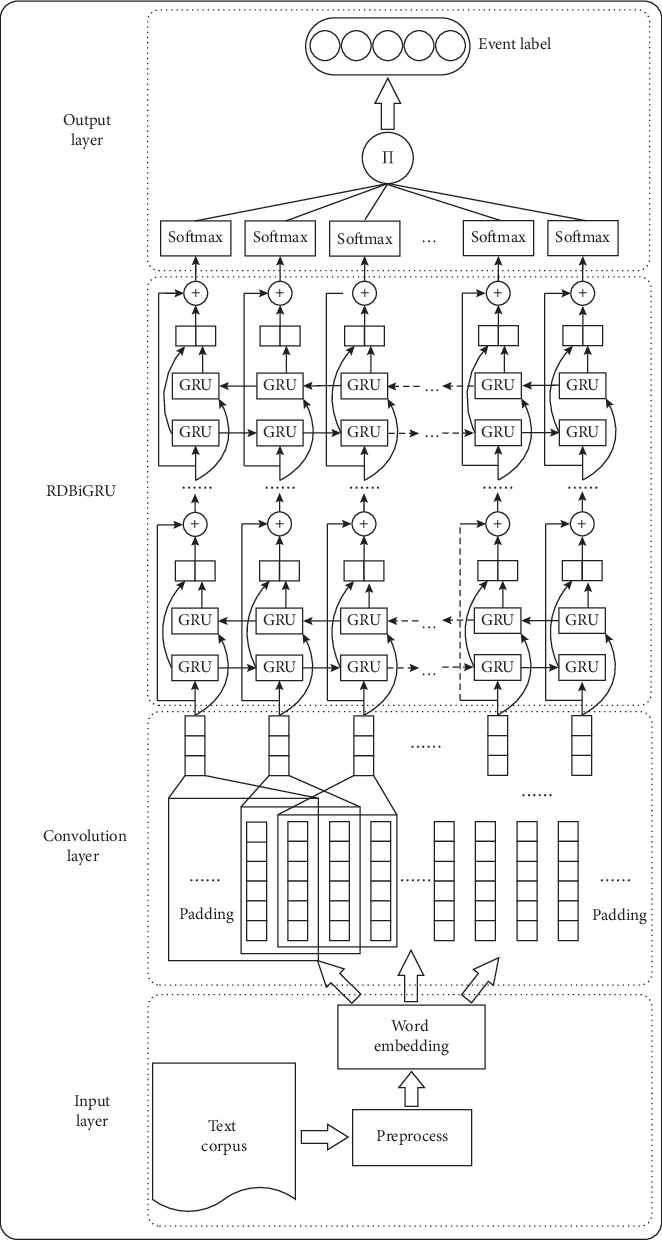
Conv-RDBiGRU model.

Firstly, the original data text is preprocessed and the word is vectorization, in the task of event recognition. Then, the word embedding matrix is input, and the corresponding type label of the event is obtained through convolution operation and RDBiGRU. Lastly, the emergency event recognition result is output. The design of the model takes into account that convolutional neural network is good at extracting *n*-gram features of local information, but the obtainment of global information features is obviously insufficient, while recurrent neural network is just opposite. Therefore, a joint model is constructed to combine with the advantages of the two neural networks. Considering that the train time of the feature learning framework is long and difficult to converge in recurrent neural network. Gradient dispersion, local optimum, and overfitting are easy to occur when the layer counts of recurrent neural network is deepened to extract deeper context semantic information. So, residual network structure is introduced to integrate an identity mapping value into output of every layer recurrent neural network. The mapping value is the current layer of input information. This can introduce more reference information to the output data, make up the influence of vanishing gradient along with the network deepened, and reduce the influence of information loss caused by the computation of hidden neurons, so as to enhance the robustness of the model and improve the effect of emergency event recognition. The emergency event recognition based on the Conv-RDBiGRU model proposed in this paper includes four parts: input layer, convolution layer, RDBiGRU, and output layer, as shown in Figure 4.

### 3.1. Input Layer

In this paper, jieba word segmentation tool was used to conduct word segmentation processing on the Text Corpus, and stop words were removed from the processed text. In deep learning, input of image data is the matrix of fixed size in advance, but text corpus data is different from the two-dimensional image data. The text size is not fixed, so the length of statement needs fixed-length processing. Let the length of the input sequence be *m* words. Segmentation text with more than *m* words intercepts front *m* words, and segmentation text with less than *m* words pad to *m* words.

The preprocessed text adopts Word2Vec model to train for obtaining the word embedding vector. The model uses the trained word vector to replace the traditional one-hot encoding method, which can avoid the problem of sparse vector space and dimension disaster, thus transforming the vector space into a low-dimensional dense form. The training method adopts the Skip-Gram model to obtain *n*-dimensional word vector *v*_*t*_ ∈ *R*^*n*^(*t* = 1, 2,…, *m*), so the text word vector matrix is *V*=[*v*_1_, *v*_2_,…, *v*_*m*_], where *V* ∈ *R*^*m×n*^.

### 3.2. Convolution Layer

**Figure 5:**
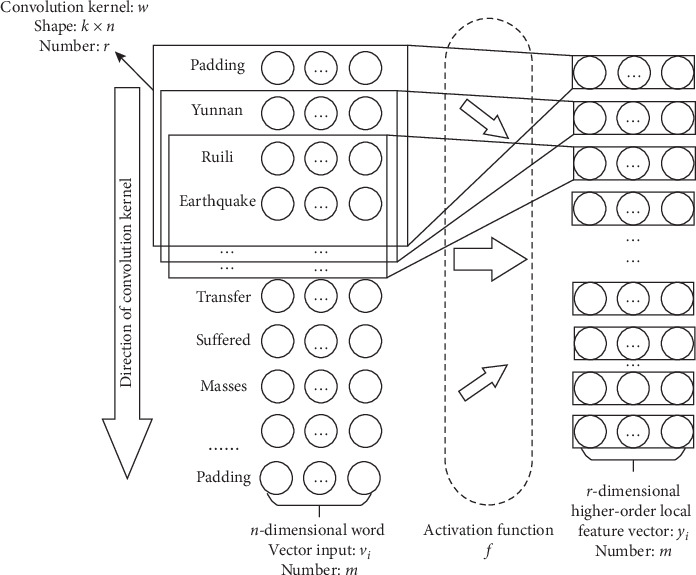
Convolution operation for extracting local features.

In the task of event recognition, the operation of the convolution layer is planned to obtain the word vector local features by sliding the convolution kernel on the word sequence and then to express them as the form of higher-order information features. The process of extracting word vector features by convolution operation is shown in Figure 5.

Let the input word vector (Yunnan, ruili, earthquake…) in sentence be expressed as *V*=[*v*_1_, *v*_2_,…, *v*_*m*_], where *v*_*i*_ ∈ *R*^*n*^ (*i* = 1, 2,…, *r*). In order to obtain word-level features, number of convolution kernel *w* is selected *r*. The structure of the *w* is *k* × *n*, namely, the matrix of *k* rows and *n* columns. Therefore, the word vector through convolution operation can be expressed as follows:

(1) $$\begin{array}{c} y_{i}=f\left(w*V^{\prime }+b\right), \end{array}$$

where the “∗” operator represents the multiplication of corresponding elements in matrix. *V*′ represents the *k* × *n* matrix composed of the word sequence vector [*v*_1_, *v*_2_,…, *v*_*k*_] in *V*, *b* is the offset vector of *n* dimension, and *f* is the nonlinear activation function. Since the convolution operation needs to input sentences of fixed length, the input sentences are padded or intercepted, while the whole sentence length remains unchanged after convolution operation. The convolution kernel is used to successively scan the word vector for obtaining the word vector local features of the whole sentence:

(2) $$\begin{array}{c} F=\left[y_{1},y_{2},\ldots ,y_{m}\right], \end{array}$$

where *F* ∈ *R*^*m×r*^, *y*_*t*_ (*t* = 1, 2,…) represents the local feature vector that the word vector goes through convolution kernel computation and is activated by activation function. The local feature of word level is taken as the input of RDBiGRU at the next layer.

### 3.3. RDBiGRU

**Figure 6:**
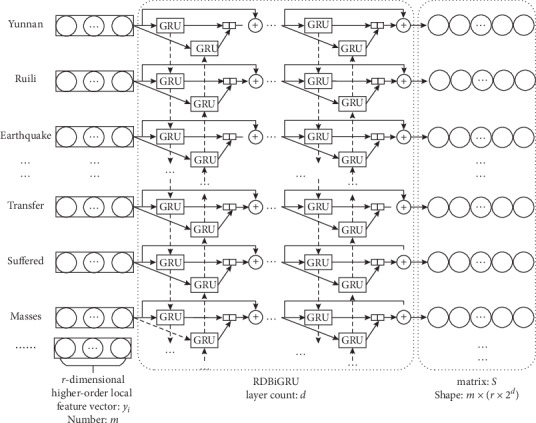
RDBiRU extracts higher-order contextual semantic features.

In event recognition tasks, operation process of RDBiGRU is to input local features vector matrix into recurrent network neural units, and then each layer obtains the information features of corresponding moment by transmission of forward and backward, and at the same time, the information features combine with the residual structure to transmit the next layer. Finally, results output higher-order features of each time sequence through feature extraction of layers. The main goal is to capture the deeper contextual semantic features of the whole text. The process is shown in Figure 6.

**Figure 7:**
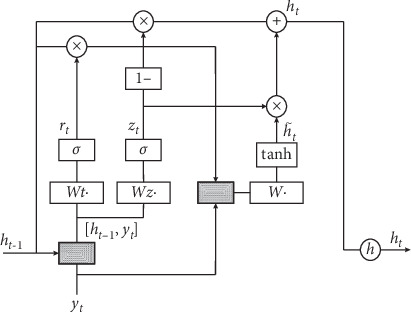
GRU unit structure.

In this paper, the recurrent neural network unit adopts GRU. The GRU simplifies the LSTM structure. It merges the input gate and the forget gate into the update gate. The structure is simpler when it reduces a gate, as shown in Figure 7.

The steps for RDBiGRU to capture deeper contextual semantic features are as follows:

1. The local feature vector *y*_*t*_ is input into the GRU unit and connected with sequence information *h*_*t*−1_ of previous time on the hidden layer. Update signal *z*_*t*_ and reset signal *r*_*t*_ are obtained through the operation of the update gate and reset gate:

(3) $$\begin{array}{c} \text{Update gate}:z_{t}=\sigma \left(Wz\cdot \left[h_{t-1},y_{t}\right]\right),\\ \text{Reset gate}:r_{t}=\sigma \left(Wr\cdot \left[h_{t-1},y_{t}\right]\right). \end{array}$$

(2) Determine the importance of h_t-1_ for candidate memory units by *r*_*t*_ ∗ *h*_*t*−1_ value. The *r*_*t*_ ∗ *h*_*t*−1_ is connected with *y*_*t*_. Candidate memory unit ${\tilde{h}}_{t}$ is obtained by weights *w* and function activation tanh.
  Candidate memory unit:

(4) $$\begin{array}{c} \tilde{h_{t}}=\tanh\left(W\cdot \left[r_{t}*h_{t-1},y_{t}\right]\right). \end{array}$$

(3) Determine how much weight the *h*_*t*−1_ will possess to transmit the next state by calculation of *z*_*t*_, *h*_*t*−1_, and ${\tilde{h}}_{t}$, and the memory unit *h*_*t*_ of the current state is obtained.
  Current memory unit:

(5) $$\begin{array}{c} h_{t}=\left(1-z_{t}\right)*h_{t-1}+z_{t}*\tilde{h_{t}}. \end{array}$$

(4) The abovementioned steps are, respectively, performed for forward GRU and backward GRU neural network units, so BiGRU is obtained. The ${\overset{⟶}{h}}_{t}$ is obtained by using forward GRU to calculate the above feature information at time *t*, while ${\overset{\leftarrow }{h}}_{t}$ is obtained by using a backward GRU to calculate the following feature information at time *t*. Thus, the output feature information can be obtained as *o*_*t*_ = [${\overset{⟶}{h}}_{t}$, ${\overset{\leftarrow }{h}}_{t}$] at this time. While *o*_*t*_ ∈ *R*^*r*×2^*h*^^ (*t* = 1, 2,…, *m*), *y*_*t*_ is convoluted through *r* × 2 × *h* convolution kernels whose shape is 1 × *r* to become a vector *y*_*t*_′, and *y*_*t*_′ ∈ *R*^1×(*r*×2×*h*)^.
(5) Let the current network layer be *h*. Add *o*_*t*_ and *y*_*t*_′ to get *o*_*t*_′. Connect *o*_*t*_′ of each moment to form the feature vector matrix *O* = [*o*_1_′, *o*_2_′,…, *o*_*m*_′], and take the matrix *O* as the input of next layer BiGRU.
(6) Let the network stack depth be *d*. Finally, result output deep contextual semantic feature *S* through recurrent neural network stack of *d* layer counts, and it takes matrix *S* as the input of the next layer.

Where *w*_*z*_, *w*_*r*_, and *w* represent weights; *σ* and tanh is the activation function; “∗” operator represents matrix elements multiplication; “” represents vector concat; *O* ∈ *R*^*m*×(*r* × 2^*h*^)^, *S* ∈ *R*^*m*×(*r* × 2^*d*^)^.

### 3.4. Output Layer

The specific operation steps of the output layer are as follows:

1. The output result *S* of the previous layer was calculated by activating function softmax to, respectively, obtain output features of corresponding time, and the probability distribution of event type *P* is obtained through calculation:

(6) $$\begin{array}{c} P=\prod_{t=1}^{n}\frac{\exp\left(s_{t}w_{fc}+b_{fc}\right)}{\sum \exp\left(s_{t}w_{fc}+b_{fc}\right)}. \end{array}$$

  While *w*_*fc*_ represents weight, *b*_*fc*_ represents offset vector; *w*_*fc*_ ∈ *R*^(*r*×2^^^*d*)×*c*^, *b*_*fc*_ ∈ *R*^*c*^, *P* = [*p*_1_, *p*_2_,…, *p*_*i*_,…, *p*_*c*_](*i* ∈ [1, *c*], *c* is the class number of emergency event).
(2) In the process of training, the crossentropy cost function is adopted as the loss function. Let the learning rate be LR and loss value be loss. The optimizer Adam is used to perform stochastic gradient descent:

(7) $$\begin{array}{c} \text{loss}=\text{average}\left(-\Sigma \text{label}*tf\cdot \log\left(p_{i}\right)\right),\\ \text{AdamOptimizer}\left(LR\right)\cdot \text{minimize}\left(\text{loss}\right). \end{array}$$

(3) The arg_max function is used in the process of model train and test, and by using this function, the index value of maximum probability *p*_*i*_ is obtained in the probability distribution of event type *P*, and the index value is matched with the actual label of the event type:

(8) $$\begin{array}{c} \text{label}=\arg\_\max\left(p_{i}\right). \end{array}$$

## 4. Experiments and Discussion

In order to validate the effectiveness of the Conv-RDBiGRU model proposed in the emergency event recognition and the recognition result is superior to the method of other model, multiple comparative experiments are performed in this paper, including hyperparameters adjustment to optimize model, comparison with different emergency event recognition models in other papers, and test in different datasets. The analysis of event recognition results adopts a common method for performance evaluation, and the standard is the same as references [12–19, 25–36], including Precision, Recall, and *F*-value.

### 4.1. Emergency Event Dataset

The Corpus of this paper adopts the CEC (Chinese Emergency Corpus) established by the semantic intelligence laboratory of Shanghai university, and the data obtained through the web crawler. Corpus data all derive from news reports of emergency event on the Internet and we-media data, which contain five types of emergency events: earthquake, fire, traffic accident, terrorist attack, and food poisoning. The total number of CEC is 332 texts. The data obtained by the crawler is 48,267 texts, and they add up 43,851 by cleaning duplicated texts. In this paper, two kinds of datasets are, respectively, conducted to experiment and randomly selected train set, validation set, and test set. These datasets are divided into 7 : 2 : 1.

### 4.2. Train Stage

In the train stage, the specific operation steps are as follows:

- Open source corpus is used in this paper and the original text corpus is XML format, namely, extensible markup language. Redundant XML elements in the text corpus are cleaned through regular expression for extracting main body of the text. Through jieba word segmentation tool, the text corpus is segmented, and redundant stop words are removed from the text after word segmentation, such as structural particles, “time,” “date,” and punctuation.
- Word2Vec model is adopted to train the preprocessed text, and Skip-Gram is adopted to obtain the word vector space of *n*-dimension vector, representing each word segmentation. Count the number of word segmentation. If the word segmentation text is more than *m* words, the text will be intercepted, and if the word segmentation text is less than *m* words, the text will be filled by using the zero vector. Thus, the input word vector matrix is *m* × *n* two-dimensional matrix *V*.
- Set *r* convolution kernel *w* whose shape is *k* × *n* matrix. Input matrix *V* becomes *m* × *r* matrix after convolution operation, and the next step gets feature matrix *F* by using nonlinear activation function *f*.
- Let stack depth of RDBiGRU network be *d*, current number of hidden layers be *h* (*h* = 1, 2,…, *d*), and the number of neuron nodes be r × 2^*h*−1^ in the GRU hidden layer. *F* Matrix was taken as the input of RDBiGRU layer to obtain *m*×(*r* × 2^*d*^) feature vector matrix *S*.
- Types of emergency event are earthquake, fire, traffic accident, terrorist attack, and food poisoning. Therefore, the number of types is set as *c*, *c* = 5.
- Crossentropy cost function is adopted as loss function to obtain loss value and let the learning rate LR be 1*e* − 3. The Adam optimizer is adopted to perform stochastic gradient descent for minimizing loss value so that the whole model can be trained to convergence.

### 4.3. Test Stage

In the test stage, the specific operation steps are as follows:

- Data preprocessing and word embedding vectors are the same as in the train stage. The processed test set is input into the Conv-RDBiGRU model that has been trained to converge.
- Through operation of convolution layer and RDBiGRU and activation of softmax function, the prediction probability value *P* of the emergency event type is obtained.
- Through arg_max function operation, the index value of maximum probability *p*_*i*_ in event type label vector *P* is matched with the actual type label of emergency event, namely, label = arg_max(*p*_*i*_), and then the match result is output.

### 4.4. Hyperparameter Determination

After word vector train, the optimal classification model needs to be determined by hyperparameter adjustment. The hyperparameters are different from weight parameters of model itself and cannot be optimized by the method of gradient descent. The different selection of these parameters will greatly affect the whole performance of the model. In this paper, the word vector dimension, Dropout value, stack layer number of residual network, and Epoch times are the hyperparameters to be adjusted. The optimal hyperparameters will be determined through experiments discussion and analysis in the crawling dataset.

#### 4.4.1. Word Vector Dimension

In order to test the influence of different word vector dimensions on the recognition results for the Conv-RDBiGRU model, Word2vec is used to, respectively, train the word vectors of 50, 100, 150, 200, 250, and 300 dimensions, and these vectors of different dimensions are taken as input in the preprocessing. The comparative experimental results are shown in Table 1.

As can be seen from Table 1, different dimensions of the word vector have a certain impact on the event recognition rate and performance of model. When the word vector dimension is 200, recognition effect of the model is the best and the *F*-value reaches 73.34%. However, with the continuous increase of dimensions, the model has a downward trend in the evaluation index. It indicates that the dimension is too high so that word vector space of the text is too large, and the feature matrix becomes sparse, which reduces the generalization ability of the model and results in the decrease of the recognition effect. Therefore, the word vector dimension is set as 200 in this paper.

#### 4.4.2. Dropout

**Figure 8:**
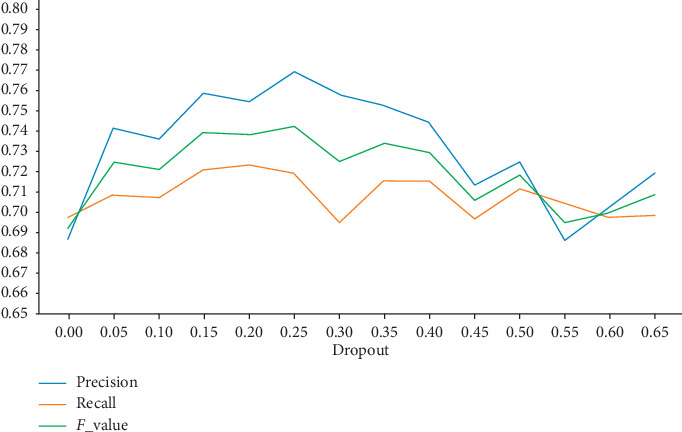
Influence of different dropout values.

In order to illustrate the influence of the Dropout value on the recognition results for the Conv-RDBiGRU model, different Dropout values are obtained in neural network processing and corresponding results were obtained in the test set for comparison, as shown in Figure 8.

As can be seen from Figure 8, when the Dropout value is 0.15–0.25, the model achieves a good recognition effect. While, when the Dropout value exceeds 0.25, the whole performance of model presents a downward trend. In deep neural network, the application of Dropout can reduce the interdependency between nodes in the learning process through randomly zeroing the hidden layer's partial weights or outputs, so as to realize the regularization of neural network, reduce the structural risk, and solve the problem of overfitting and vanishing gradient to a certain extent. The set should be reasonable; otherwise, it will reduce the model performance. Therefore, the Dropout value is set as 0.25 to achieve better event recognition performance.

#### 4.4.3. Stacked Counts of ResNet

In order to compare the effects of different network structures on the recognition results for the Conv-RDBiGRU model, the number of stacked layers is differently evaluated in deep residual network structure, and the recognition effects of corresponding network structures are obtained, as shown in Table 2.

As can be seen from Table 2, when the stack depth is 4 layers, the model achieves the best recognition effect. When the stack depth continues to deepen, the recognition performance of the model starts to decline. It indicates that the stack depth of residual network should be moderate, and if the number of stack layers is too deep, it does not necessarily optimize the model. Therefore, the stack depth of the residual network is set as 4 layers in this paper.

#### 4.4.4. Epoch

**Figure 9:**
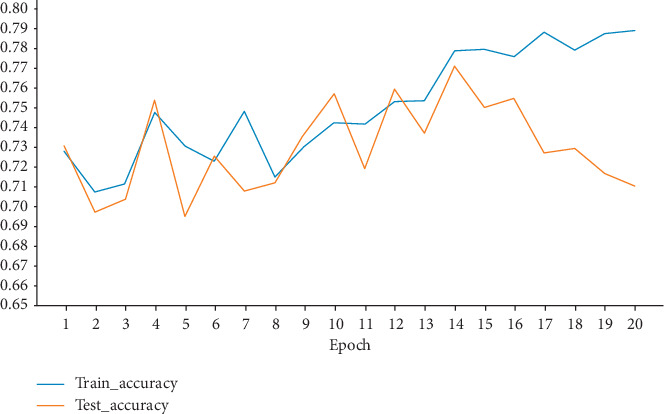
Influence of different Epochs.

In the process of model learning, all samples in the train set are trained once, which means that recognition accuracy of the model is obtained about the current corresponding Epoch.  Figure 9 shows situation changes of relevant indexes in the model when the Epoch is 1–20.

As can be seen from Figure 9, when the Epoch is 14, the recognition accuracy of the test set reaches the optimal value. When the Epoch is more than 14, although the accuracy of the model in the train set is still improved, the accuracy in the test set has a significant trend of decline, indicating that the model has been overfitting. The number of iterations needs to be adjusted manually in deep neural network train and the number of iterations may be varied depending on the task being processed. On the one hand, if number of iterations is too little, the model will not be converged to a local minimum. The model will be resulting in underfitting. On the other hand, if number of iterations is too many, the train time of model will be prolonged, and model will face problem of overfitting, so as to lose its generalization. Therefore, the Epoch value was set as 14 in this paper to achieve better event recognition performance.

### 4.5. Result Analysis

In this paper, multiple experiments are performed, including CNN, GRU, BiGRU and joint model Conv-BiGRU, CNN-BiGRU, Conv-DBiGRU, and Conv-RDBiGRU. The results of emergency event recognition are compared with different models in other papers. The experimental comparison results in CEC are shown in Table 3.

Because CEC which is structured processing is in the XML language format, the text features are still obvious and easier to extract and train after preprocess so that it relatively weakened superiority of the convolution layer in extraction aspect of partial feature. It is the loss of generality. On the contrary, CEC has little data, which may lead to insufficient model training and cannot reflect the generalization ability of the model, so the experiment needs to perform comparison experiment for unstructured data information that is crawled on we-media of the Internet. The experimental comparison results are shown in Table 4.

As can be seen from the experimental results, the traditional machine learning method, SVM model, was adopted to extract events in [37] of Tables 3 and 4, which achieved high precision but low recall. In terms of the effect of event recognition, the deep learning method is more effective than the traditional machine learning method, and the overall *F*-value is better than SVM, indicating that deep learning can extract more abstract features to perform train. CNN adopts convolutional neural network to perform event recognition, and GRU adopts unidirectional gated recurrent unit. The *F*-value of GRU is slightly higher than CNN, indicating that the context information captured by GRU is more conducive to the emergency event recognition. The effect of the Conv-DBiGRU model in Tables 3 and 4 is not good, which indicates that to simply deepen the overall depth of the network does not necessarily improve recognition effect of events, but the effect obtained in Table 4 is relatively better than that in Table 3, which indicates that fitting effect of deep neural network trained in a larger dataset is better. In Table 3, Ma et al. [38] adopt the BiGRU model and the *F*-value reached 70.00%, which is nearly 2% higher than that of GRU, indicating that bidirectional recurrent neural network by capturing the features of context information is better than unidirectional that in performance of emergency event recognition. In Table 4, the recognition performance of Conv-BiGRU is better than that of CNN-BiGRU, indicating that, in a larger dataset, the pooling operation of model can be abandoned to obtain a better recognition performance. In Table 3, the transfer model is better than Doc2EDAG and DCFEE, but in Table 4, the *F*-value of Doc2EDAG is slightly higher than transfer, indicating that the method using transfer learning has better recognition effect. Doc2EDAG has a higher *F*-value than DCFEE, indicating that improvements are made on the basis of DCFEE so that multievent sentences with just a sequence tagging model can be processed reasonably to obtain better recognition result. In LEAM model, a method is proposed that joints embedding of words and labels for text classification. In Table 3, LEAM achieves a good recognition effect. It may be that the CEC dataset is a structured public dataset and the attention mechanism is incorporated into the LEAM model, which improves the interpretability of structured information so that it achieves higher *F*-value, but the precision is not high. In Table 4, the unstructured we-media data is used as the dataset. Conv-RDBiGRU can extract more abstract semantic features through deep neural network architecture. After repeated training, better recognition results are obtained. On the basis of stacked bidirectional recurrent neural network Conv-DBiGRU, Conv-RDBiGRU introduces the residual network structure, which makes original input information integrate into output information processed by the hidden neuron and makes more abundant features be learned at gradient update. For deep neural network, the problem of vanishing gradient can be alleviated in certain extent. Compared with Conv-DBiGRU, the performance of Conv-RDBiGRU is greatly improved and a higher *F*-value is obtained.

The Conv-RDBiGRU joint model proposed in this paper performs the comparison with other methods. The precision and recall all achieved good result, and *F*-value is superior to other methods, indicating that, in capture of event local features, extraction of deep context semantic feature, train and generalization of whole model, the neural network model combined with convolution operation, and recurrent and residual structure can achieve good improvement effect. It improves the performance of Chinese emergency event recognition.

## 5. Conclusion and Future Work

In the task of Chinese emergency event recognition, Conv-RDBiGRU neural network model is proposed in this paper. Firstly, text corpus is preprocessed by word segmentation and stop words processing and uses word embedding to form the matrix of word vectors. Deep context information features are extracted through DBiGRU, and at the same time, the residual structure is introduced into DBiGRU, which integrates an identity mapping value into the output features that is extracted by every layer neural network, and the model has more complete reference information in gradient descent in model train, which can learn more abundant feature information, so as to reduce the influence of information loss because of calculation of hidden layer neurons, and alleviate the problem of vanishing gradient during model train. In this way, the model has better feature learning ability and generalization. Experimental results show that this method is feasible and effective for emergency event recognition.

Considering that the proposed method may be tested with a larger dataset, semantic information of text can be further expanded, and the model lacks the interpretability of information features that possess different levels of importance. The next step will test with a larger dataset, integrate more semantic features into the research to enrich the dimension of word embedding vectors in the input layer, and try to add other network structures to improve the interpretability of the model for different information features, and enhance the generalization of model, so as to improve the performance of emergency event recognition.

## Figures and Tables

**Table 1:** Influence of different word vector dimensions.

Dimensions	*P* (%)	*R* (%)	*F* (%)
50	68.54	70.39	69.45
100	72.45	71.14	71.79
150	74.13	70.84	72.45
**200**	**75.25**	**71.53**	**73.34**
250	70.21	69.71	69.96
300	71.33	69.65	70.49

**Table 2:** Influence of different stack depths.

Depth	*P* (%)	*R* (%)	*F* (%)
2	75.81	72.06	73.89
3	74.69	71.76	73.20
**4**	**76.93**	**71.98**	**74.37**
5	74.42	71.51	72.94
6	71.89	69.83	70.85
7	73.01	69.40	71.16

**Table 3:** Experimental comparison results in CEC.

Model	*P* (%)	*R* (%)	*F* (%)
SVM [37]	79.30	59.90	63.70
Conv-DBiGRU	72.31	63.51	67.62
CNN	72.73	64.00	68.09
GRU	69.70	66.67	68.15
DCFEE [28]	68.07	70.85	69.43
BiGRU [38]	71.10	69.00	70.00
Conv-BiGRU	73.02	69.70	71.32
Doc2EDAG [33]	73.49	70.31	71.87
Transfer [34]	74.09	70.48	72.24
CNN-BiGRU	74.24	71.01	72.59
Conv-RDBiGRU	78.79	69.33	73.76
LEAM [35]	71.08	79.72	75.15

**Table 4:** Experimental comparison results in we-media data.

Model	*P* (%)	*R* (%)	*F* (%)
SVM [37]	78.23	54.51	64.25
GRU	71.02	61.06	65.67
CNN	71.72	65.58	68.52
DCFEE [34]	72.46	69.05	70.72
BiGRU [38]	75.14	69.61	72.27
Transfer [28]	77.91	70.64	74.10
Doc2EDAG [33]	76.42	72.33	74.32
Conv-DBiGRU	76.86	73.41	75.10
CNN-BiGRU	78.75	72.79	75.65
LEAM [35]	73.51	81.37	77.24
Conv-BiGRU	82.04	75.45	78.60
Conv-RDBiGRU	81.36	78.15	79.72

## Data Availability

The data used to support the findings of this study have been deposited in https://github.com/shijiebei2009/CEC-Corpus.
